# The dual immunomodulatory role of B cells in tumorigenesis: mechanisms, microenvironment crosstalk, and therapeutic implications

**DOI:** 10.3389/fimmu.2025.1649812

**Published:** 2025-10-30

**Authors:** Zhuangwei Lv, Ruohao Yang, Kai Zhang, Ruihan Wang, Xiaoyu Shi, Jinhua Wu, LuLu Liu, Junna Jiao

**Affiliations:** ^1^ School of Forensic Medicine, Xinxiang Medical University, Xinxiang, Henan, China; ^2^ School of Basic Medical Sciences, Xinxiang Medical University, Xinxiang, Henan, China; ^3^ School of Junji College, Xinxiang Medical University, Xinxiang, Henan, China; ^4^ Xinxiang Engineering Technology Research Center of immune checkpoint drug for Liver-Intestinal Tumors, Xinxiang Medical University, Xinxiang, Henan, China

**Keywords:** B cells, tumor microenvironment, immunotherapy, tumor immunity, tertiary lymphoid structure

## Abstract

B lymphocytes exhibit a multifaceted and context-dependent role in tumor biology, acting as both promoters and suppressors of malignancy through dynamic interactions within the tumor microenvironment (TME). This review synthesizes current evidence on the dual functions of B cells in tumor immunity, highlighting their capacity to orchestrate antitumor responses via antigen presentation, antibody-dependent cytotoxicity, and tertiary lymphoid structure (TLS)-mediated T cell activation, while paradoxically driving immunosuppression through regulatory B cells (Bregs), pro-angiogenic signaling, and immune checkpoint modulation. Key mechanisms include TLS formation, which enhances cytotoxic T cell priming and correlates with improved immunotherapy outcomes, and Breg-mediated secretion of IL-10/TGF-β, which fosters T cell exhaustion and myeloid-derived suppressor cell recruitment. Tumor-type specificity is evident: TLS-rich malignancies like melanoma and Non-Small Cell Lung Cancer (NSCLC) show B cell-driven immune activation, whereas pancreatic and hepatocellular carcinomas demonstrate B cell functional plasticity influenced by metabolic and epigenetic reprogramming. Therapeutically, B cell-targeted strategies—including CD20 antibodies, CAR-T cells, and B cell epitope vaccines—demonstrate efficacy in hematologic and solid tumors, yet face challenges due to subset heterogeneity and sex-specific response disparities. Emerging approaches combine immune checkpoint inhibitors (ICBs) with TLS-inducing agents or exploit B cell-derived biomarkers for personalized therapy. Future directions emphasize deciphering B cell metabolic-niche crosstalk, optimizing combinatorial regimens, and leveraging spatial multiomics to resolve functional heterogeneity. By bridging mechanistic insights with clinical translation, this work underscores B cells as pivotal regulators of tumor immunity and advocates for precision strategies to harness their antitumor potential while mitigating pro-tumor plasticity.

## 1 Introduction

Tumorigenesis represents a multifaceted biological process involving dynamic interactions between malignant cells and host immunity. While T lymphocytes have dominated tumor immunology research, emerging evidence underscores the critical yet underappreciated role of B lymphocytes within the TME (1). As integral components of adaptive immunity, B cells undergo a tightly regulated differentiation process: upon antigen encounter, naïve B cells can proliferate and differentiate into memory B cells(MBCs) or antibody-secreting plasma cells(PCs), mediating long-term humoral immunity. Beyond their canonical role in antibody production, B cells also function as antigen-presenting cells and cytokine secretors, thereby modulating both innate and adaptive immune responses (2).

Under physiological conditions, this differentiation is critically shaped by signals from the microenvironment, including cytokines, T cell help, and antigen affinity. However, in the tumor context, these precisely regulated processes are often co-opted or dysregulated. Recent advances have elucidated novel mechanisms by which B cells shape anti-tumor immunity, revealing their potential as diagnostic biomarkers and therapeutic targets (3). This evolving paradigm highlights the urgency to decipher B cell biology in malignancy, which may catalyze the development of innovative immunotherapies and prognostic tools.

## 2 Classification and functional overview of B cells

Single-cell transcriptome profiling has revolutionized B cell classification, refining it into numerous phenotypically and functionally distinct subsets beyond traditional lineages, particularly in the context of human cancers. Naïve B cells, marked by genes like TCL1A, FCER2, and IGHD, serve as primary reservoirs for antigen recognition (4). Activated B cell subsets, such as EGR1^+^ACB1, NR4A2^+^ACB2, and CCR7^+^ACB3, are characterized by CD69 and CD83 expression, reflecting early activation states (5). Germinal center (GC) B cells had relatively high expression of MKI67, which mediate somatic hypermutation(SHM) and class-switch recombination for high-affinity antibody production (6). Key newly identified subsets include atypical MBCs, expressing markers like ITGAX and FCRL5, with an exhausted phenotype and progenitor potential for extrafollicular-derived antibody-secreting cells (ASCs); tumor-associated atypical B cells, which interact with CD4^+^ T cells and predict favorable prognosis; and heterogeneous MBCs (1, 5). ASCs, including plasmablasts and multiple plasma cell subclusters, exhibit distinct tissue preferences and functional specialization in antibody secretion, with isotype shifts (e.g., IgG bias in tumors vs. IgA-dominance in adjacent tissues) driven by the microenvironment (5).

Clonal expansion characteristics, inferred from B cell receptor (BCR) repertoire and single-cell RNA sequencing (scRNA-seq) dynamics, further distinguish B cell subsets and underpin their functional roles. GC-derived ASCs, enriched in some cancers (e.g., colon adenocarcinoma), show high clonal diversity, extensive SHM, and preferential class-switch recombination to IGHA1/2, reflecting antigen-driven affinity maturation (5). In contrast, EF-derived ASCs, dominant in others (e.g., liver hepatocellular carcinoma), exhibit oligoclonal expansion with lower SHM, limited class-switch recombination, and enrichment of IGHM/IGHG4, linked to polyreactive/autoantibody production (5). Atypical MBCs display moderate clonal overlap with EF-derived ASCs, independent of GC pathways, and their expansion correlates with immunosuppressive microenvironments. tumor-associated atypical B cells and IgG^+^ ASCs undergo robust clonal expansion in tumors, indicative of antigen-driven activation (1). GC B cells show frequent clonal sharing, supporting iterative differentiation into MBCs and ASCs with accumulated SHM to enhance antibody affinity. These clonal dynamics, coupled with subset-specific interactions (e.g., tumor-associated atypical B cells with CD4^+^ T cells via MHC-II), shape B cells’dual roles in humoral immunity and T cell regulation, with implications for antitumor immunity, immune evasion, and immunotherapy response (1).

## 3 Basic characteristics of B cells in the tumor microenvironment

While the classification of B cell subsets provides a foundational understanding of their functional diversity, their spatial distribution and organizational states within the TME further dictate their roles in tumor immunity.

The TME, a multicellular ecosystem comprising malignant cells, stromal fibroblasts, vascular networks, and immune populations, exhibits significant spatial heterogeneity in B lymphocyte distribution (7). B cell infiltration patterns demonstrate tumor-type specificity and stage-dependent variation: while malignancies such as melanoma and triple-negative breast cancer display dense B cell infiltrates that frequently organize into TLSs– organized lymphoid aggregates supporting coordinated antitumor immunity – other tumors like pancreatic adenocarcinoma show minimal B cell recruitment (8). This spatial heterogeneity stems from tripartite regulatory mechanisms: tumor-intrinsic features (e.g., mutational burden, chemokine secretion profiles); microenvironmental constraints (hypoxia, extracellular matrix remodeling), and host immunological status (peripheral B cell repertoire diversity, pre-existing antitumor memory) (9). Notably, TLS formation correlates with enhanced cytotoxic T cell priming and improved clinical outcomes, highlighting the functional implications of B cell spatial organization in the TME (10–13).

## 4 The dual role of B cells in tumor immunity

### 4.1 Antitumor mechanisms

#### 4.1.1 Antigen presentation and coordinated T cell activation

**Figure 1:**
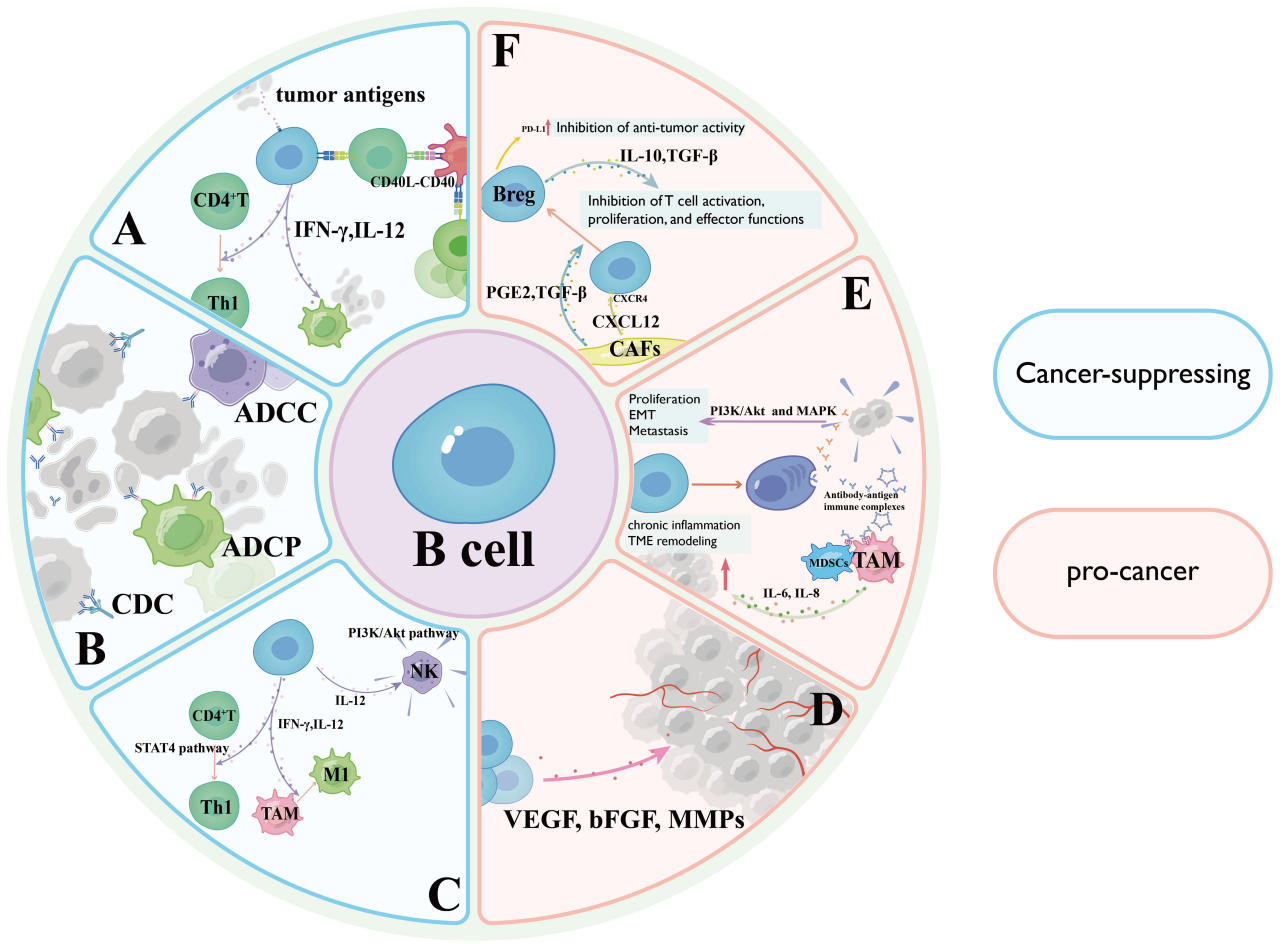
Diagram illustrating the dual roles of B cells in cancer, highlighting cancer-suppressing mechanisms in light blue and pro-cancer mechanisms in pink. **(A)** Antigen Presentation and Coordinated T Cell Activation Mechanisms; **(B)** Antibody-Dependent Antitumor Effector Mechanisms; **(C)** Cytokine Network and Tumor Microenvironment Reprogramming; **(D)** Secretion of vascular endothelial growth factor (VEGF), basic fibroblast growth factor (bFGF), and matrix metalloproteinases (MMPs) by activated B cells stimulates endothelial cell proliferation and migration through VEGF receptor 2 (VEGFR2) signaling; **(E)** tumor-infiltrating B cells may paradoxically promote oncogenesis through antibody-dependent pathways; **(F)** Regulatory B cells (Bregs) exert immunosuppressive effects through the secretion of potent immunosuppressive cytokines.

B lymphocytes orchestrate antitumor responses through multifaceted mechanisms, most notably via professional antigen presentation (7). By internalizing tumor-associated antigens (TAAs) and processing them through MHC-II pathways, B cells present immunogenic peptides to CD4^+^ T cells via TCR engagement, crucially licensing dendritic cells for subsequent CD8^+^ T cell priming through CD40-CD40L costimulatory interactions (14). Mechanistically, this antigen cross-presentation cascade amplifies cytotoxic T lymphocyte activation, proliferation, and tumor infiltration while promoting immunological memory formation (14). Experimental evidence from B cell-deficient murine models reveals profound impairment of T cell effector functions and tumor control, underscoring the essential role of B cells in coordinating adaptive immunity (15). Furthermore, B cell-derived cytokines including IL-12 and IFN-γ enhance Th1 polarization and macrophage tumoricidal activity, creating a self-reinforcing antitumor immune loop (16) (**Figure 1A**).

#### 4.1.2 Antibody-dependent antitumor effector mechanisms

Upon antigen-specific activation, tumor-reactive B cells differentiate into antibody-secreting PCs that generate high-affinity immunoglobulins targeting tumor-associated surface markers (17). These antibodies execute multifaceted antitumor effects through three principal mechanisms: Antibody-dependent cellular cytotoxicity mediated by Fcγ receptor activation on natural killer cells and macrophages; Complement-dependent cytotoxicity initiated through C1q binding and membrane attack complex formation; Opsonization-enhanced phagocytosis via Fc receptor engagement on myeloid cells (18). Clinically, this paradigm is exemplified by CD20-targeting rituximab in B cell lymphomas, where antibody-mediated B cell depletion achieves durable remissions (19). Furthermore, certain antibodies disrupt oncogenic signaling by competitively inhibiting growth factor receptors (20) (**Figure 1B**).

#### 4.1.3 Cytokine network and tumor microenvironment reprogramming

Within the TME, B lymphocytes orchestrate coordinated antitumor immunity through cytokine-mediated crosstalk (21). Secretion of interleukin-12 and interferon-γ enables B cells to potentiate T cell cytotoxicity by driving Th1 polarization through signal transducer and activator of transcription4-dependent transcriptional programming, while simultaneously enhancing natural killer cell degranulation capacity via PI3K/Akt pathway activation (21). Furthermore, interferon-γ reprograms tumor-associated macrophages (TAMs) toward an immunostimulatory M1 phenotype characterized by heightened phagocytic activity and inducible nitric oxide synthase expression, effectively reversing immunosuppressive TME conditions (22). Clinical correlative studies demonstrate that B cell-derived interleukin-12/interferon-γ levels correlate with improved cytotoxic lymphocyte infiltration and survival outcomes in melanoma and colorectal carcinoma (23) (**Figure 1C**).

A Antigen Presentation and Coordinated T Cell Activation Mechanisms; B Antibody-Dependent Antitumor Effector Mechanisms; C Cytokine Network and Tumor Microenvironment Reprogramming; D Secretion of vascular endothelial growth factor(VEGF), basic fibroblast growth factor, and matrix metalloproteinases by activated B cells stimulates endothelial cell proliferation and migration through VEGF receptor 2 signaling; E tumor-infiltrating B cells (TIL-Bs) may paradoxically promote oncogenesis through antibody-dependent pathways; F Bregs exert immunosuppressive effects through the secretion of potent immunosuppressive cytokines.

#### 4.1.4 The links between TLS maturity and B cell function

The maturity of TLSs directly influences the efficiency of B cell activation, antibody secretion, and T cell helper functions. The specific mechanisms are as follows:

##### 4.1.4.1 Immature TLS and B cell activation

Immature TLS (such as primary follicles) typically lack GCs, resulting in distinct B cell differentiation trajectories compared to mature TLS. In this context, B cell activation relies on direct antigen stimulation; however, the affinity is relatively low, and the main antibody secreted is IgM (24, 25). Additionally, B cells in immature TLS tend to differentiate into Bregs. These Bregs inhibit immune responses by secreting cytokines like IL-10, thereby establishing an immune-tolerant environment (24, 25).

##### 4.1.4.2 Mature TLS and B cell activation

Mature TLS, such as secondary follicles, possess active GCs where B cells undergo class switching (e.g., from IgM to IgG) and affinity maturation. Here, the two-signal activation model plays a dominant role: the first signal is triggered when the BCR specifically binds to antigenic epitopes, and the second signal is provided by T cells—via the binding of CD40L to CD40 on B cells—along with T cells secreting cytokines like IL-4, which together drive B cell proliferation and differentiation into PCs and memory cells (24).

##### 4.1.4.3 Mature TLS and antibody secretion

In mature TLS, plasma cell differentiation is more efficient, and the primary antibodies produced are of the IgG class. These antibodies clear pathogens or TAAs through mechanisms such as neutralization and opsonization. Simultaneously, B cells enhance antibody affinity via SHM, contributing to the formation of immunological memory (25).

##### 4.1.4.4 Aberrant TLS and immune imbalance

Aberrantly immature TLS may contribute to autoimmune diseases (e.g., systemic lupus erythematosus) due to enhanced BCR signaling and the lack of effective co-stimulatory regulation. Conversely, excessively mature TLS might trigger tumor immune evasion. For instance, in solid tumors like breast cancer, IgG antibodies produced by high-density PCs may facilitate the masking of TAAs (24, 26).

#### 4.1.5 TLS-mediated T cell activation

**Figure 2:**
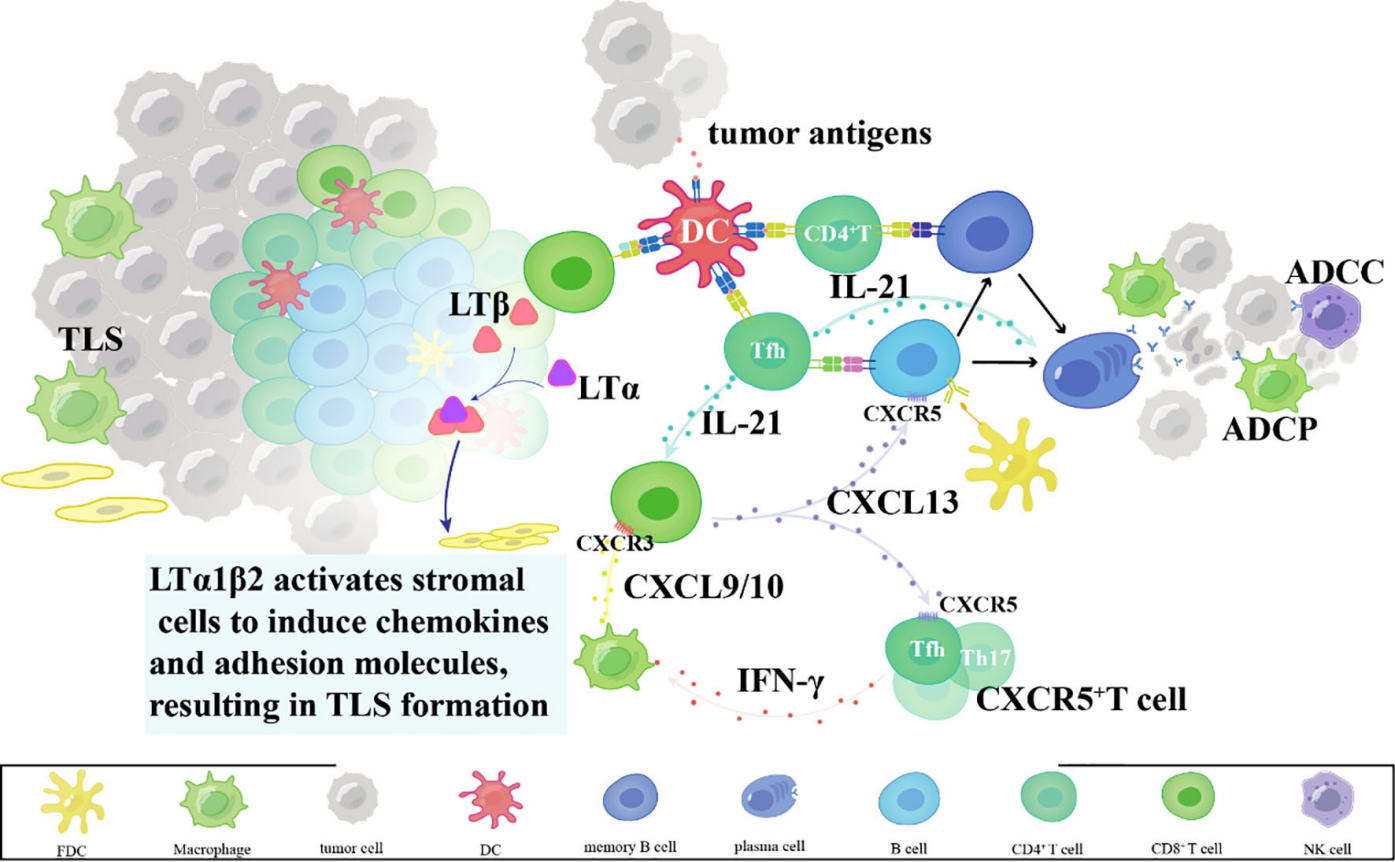
Mechanisms of TLS-mediated T cell activation and dendritic cell regulation.

Within TLSs, B cells function as professional antigen-presenting cells, internalizing and processing tumor-derived antigens for presentation via MHC class II molecules to CD4^+^ T cells (27). Concurrently, B cell-derived chemokines such as CXCL13 establish chemotactic gradients that recruit CXCR5^+^ T cell subsets – particularly follicular helper T cells and Th17 cells – into TLS niches (28–30). These recruited T cells amplify antitumor responses through dual mechanisms: IFN-γ secretion to enhance CTL tumor infiltration via CXCL9/10 induction, and IL-21 production to support B cell antibody affinity maturation (31–33). Spatial proximity within TLS thus creates an immunologically active hub for coordinated T-B cell collaboration (34, 35) (**Figure 2**).

#### 4.1.6 TLS-dependent dendritic cell regulation

B cells also modulate dendritic cell (DC) activity within TLS via cytokine-mediated crosstalk (36). Mature DCs reciprocally enhance TLS function by cross-presenting tumor antigens via MHC class I to activate CD8^+^ T cells and producing IL-12 to sustain Th1 polarization (37). This bidirectional synergy establishes a self-reinforcing loop: DC-primed T cells secrete lymphotoxin-β to maintain TLS stromal architecture, while TLS-resident DCs acquire enhanced capacity for tumor antigen uptake through Fc gamma receptor-mediated immune complex internalization (38). Such coordinated interactions transform TLS into functional equivalents of secondary lymphoid organs, enabling sustained adaptive immunity against progressing malignancies (38) (**Figure 2**).

Tertiary lymphoid structures in tumors; mechanisms of tertiary lymphoid structure formation.

While B cells can orchestrate potent antitumor immune responses through the mechanisms described above, it is increasingly evident that certain B cell subsets also contribute to immunosuppression and tumor progression. The following section discusses these paradoxical pro-tumor roles, highlighting the context-dependent nature of B cell functions in cancer.

### 4.2 Pro-tumor mechanisms

Certain B cell subsets, particularly Bregs, exert immunosuppressive effects through the secretion of potent immunosuppressive cytokines such as interleukin-10 (IL-10) and transforming growth factor-β (39, 40). These cytokines directly inhibit T cell activation, proliferation, and effector functions, facilitating tumor progression and immune evasion (40). Preclinical studies across multiple tumor models demonstrate a strong correlation between Breg infiltration, accelerated tumor growth, and poor clinical outcomes (41) (**Figure 1F**).

A subset of TIL-Bs may paradoxically promote oncogenesis through antibody-dependent pathways (5). Pathogenic autoantibodies targeting tumor-associated surface antigens can activate pro-survival signaling cascades (e.g., PI3K/Akt and MAPK pathways), thereby enhancing tumor cell proliferation, epithelial-mesenchymal transition, and metastatic dissemination (42). Furthermore, antibody-antigen immune complexes engage Fcγ receptors on myeloid-derived suppressor cells and TAMs, amplifying immunosuppressive cytokine networks (e.g., IL-6, IL-8) while triggering chronic inflammation that fosters tumor niche establishment (43) (**Figure 1E**).

B cells also contribute to tumor neovascularization through paracrine interactions within the TME (44). Secretion of VEGF, basic fibroblast growth factor, and matrix metalloproteinases by activated B cells stimulates endothelial cell proliferation and migration through VEGF receptor 2 signaling, ultimately establishing pro-angiogenic networks that sustain tumor metabolic demands and facilitate hematogenous metastasis (45) (**Figure 1D**).

Emerging evidence indicates bidirectional crosstalk between Bregs and stromal components, particularly cancer-associated fibroblasts (46). CAFs recruit B cell precursors via CXCL12/CXCR4 axis activation and induce Breg polarization through dual secretion of Transforming Growth Factor-beta(TGF-β) and prostaglandin E2 (47).

### 4.3 Molecular mechanisms governing B cell functional plasticity

The functional plasticity of B cells in the TME is principally governed by dynamic transcriptional networks. Key transcription factors play decisive roles in fate determination: B-cell lymphoma 6 maintains GC B cell identity and prevents premature differentiation, whereas B lymphocyte-induced maturation protein-1 promotes plasma cell maturation and antibody production (48, 49). Conversely, the differentiation of Bregs is driven by STAT3 activation, often induced by cytokines such as IL-10 and IL-35 within the TME (50, 51). BTB and CNC homology 2 acts as a critical repressor that preserves B cell plasticity by restraining terminal differentiation and maintaining a reversible functional state (52).

Beyond transcriptional control, epigenetic and metabolic mechanisms stabilize B cell phenotypes and enforce functional commitment. Tumor-derived signals—including hypoxia, cytokines, and nutrient scarcity—reshape the epigenetic landscape through DNA methylation, histone modifications, and non-coding RNAs, thereby locking B cells into specific transcriptional programs (53). Concurrently, metabolic reprogramming modulates B cell function; hypoxia-inducible factor-1α activation under low oxygen tension alters glycolytic flux and oxidative phosphorylation, influencing survival, proliferation, and immunoglobulin class switching.

The integration of extrinsic signals through specific receptors ultimately dictates B cell function via coordinated molecular cascades. For instance, Transforming Growth Factor-beta plus IL-21 signaling through cytokine receptors promotes Breg generation via STAT pathways, while CD40L and IL-4 engagement activates NF-κB and PI3K signaling to foster GC responses (50). These signaling networks converge to regulate the transcriptional, epigenetic, and metabolic networks described above, forming a coherent “signal–mechanism–phenotype” axis that explicates how microenvironmental cues steer B cells toward either pro-tumor or anti-tumor identities.

Given the context-dependent nature of B cell functions, it is imperative to examine how these mechanisms manifest across different cancer types, which we explore in the following section.

## 5 B cell interactions within the tumor microenvironment: crosstalk with immune and cancer cells

### 5.1 Interactions between B cells and T cells

B cells and T cells engage in bidirectional functional cooperation during antitumor immune responses (2, 54–56). As professional antigen-presenting cells, B cells prime CD4^+^ T cell activation through MHC-II-mediated tumor antigen presentation (57). This antigen-specific stimulation, complemented by co-stimulatory signals such as CD80/CD86, ensures full T cell activation.

**Figure 3:**
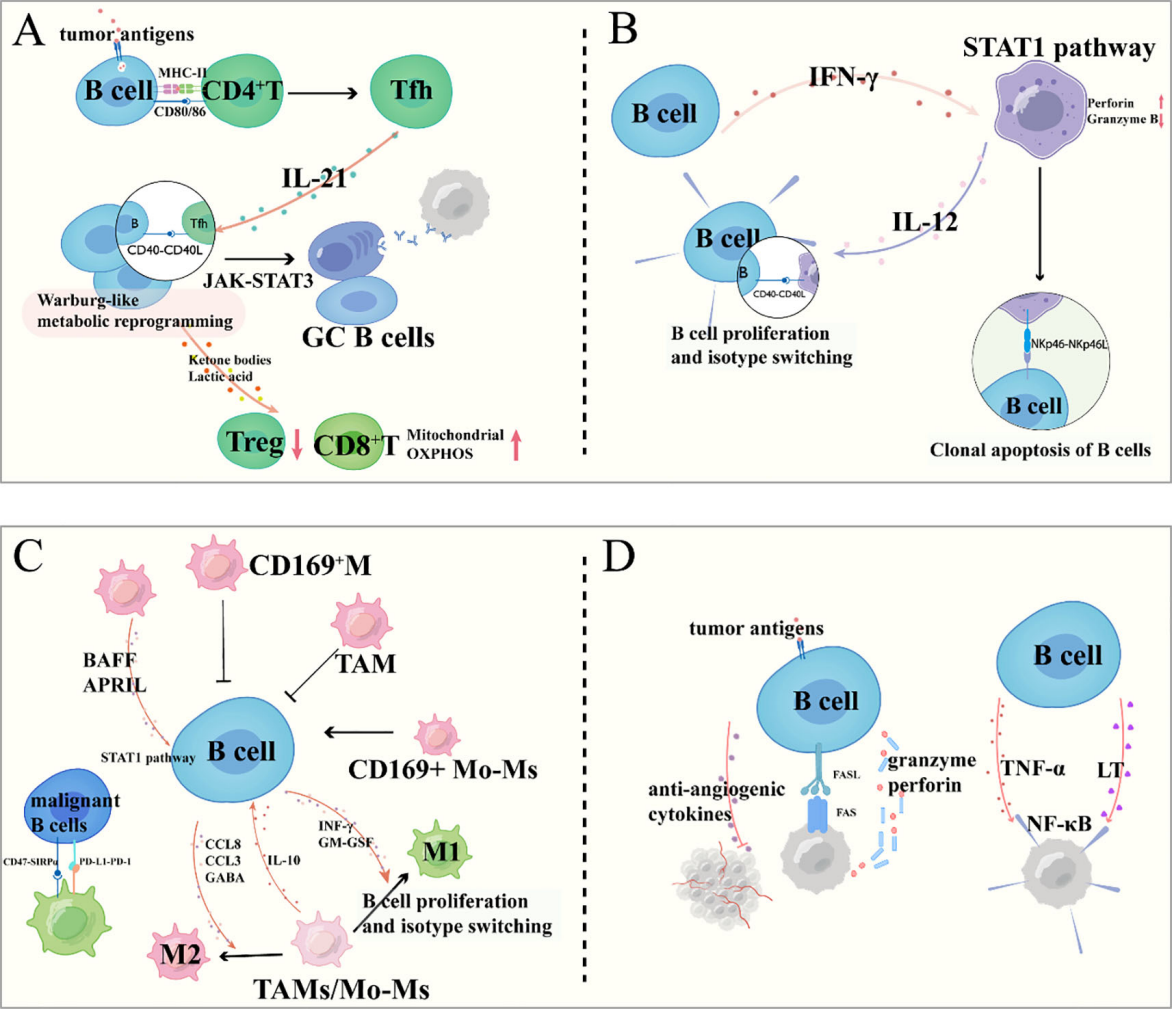
B cell interactions within the tumor microenvironment: crosstalk with immune and cancer cells. **(A)** Interactions between B Cells and T Cells; **(B)** Regulatory Dynamics between B Cells and NK Cells; **(C)** Bidirectional Crosstalk between B Cells and Tumor-Associated Macrophages; **(D)** Direct and Indirect Interactions between B Cells and Cancer Cells.

Reciprocally, activated T cells secrete cytokines such as IL-21, which drives B cell proliferation, plasma cell differentiation, and enhanced antibody production through STAT3-dependent transcriptional activation (58, 59). Tfh cells are crucial instructors of B cell fate and function (60–62). Through direct cell contact mediated by CD40L binding to CD40 on B cells, and through the secretion of cytokines—most notably IL-21—Tfh cells provide essential signals that guide B cell differentiation. IL-21 signaling activates the JAK-STAT3 pathway in B cells, leading to transcriptional upregulation of genes that promote proliferation, isotype switching, and ultimately, their differentiation into antibody-secreting PCs or GC B cells (63). This high-level cooperation is spatially coordinated within tumor-associated TLSs, which serve as organized hubs for the generation of high-affinity, tumor-specific antibodies and MBCs (64) (**Figure 3A**).

Conversely, B cells significantly influence the function and differentiation of T cells, particularly Tfh cells. By presenting antigen, B cells help sustain Tfh cell survival and functional maturation within TLS (64). Furthermore, activated B cells undergo metabolic reprogramming, increasing their glycolytic flux and glutamine metabolism. The resulting metabolic byproducts, such as lactate, can enhance mitochondrial oxidative phosphorylation in neighboring effector T cells, thereby boosting their antitumor activity, while simultaneously inhibiting the expansion of immunosuppressive regulatory T cells (65, 66).

In summary, the interaction between B and T cells is a dynamic, reciprocal relationship encompassing antigen presentation, co-stimulatory signaling, cytokine communication, and metabolic cross-talk. This coordinated network is vital for directing both cellular and humoral arms of the immune system against tumors.

### 5.2 Regulatory dynamics between B cells and natural killer cells

The interplay between B cells and NK cells exhibits dual regulatory roles in tumor immunity (7, 67). B cell-derived interferon-γ primes NK cell activation through STAT1-mediated transcriptional upregulation of perforin and granzyme B, thereby enhancing NK-mediated tumor cell lysis. Conversely, NK cells regulate B cell responses through both contact-dependent and cytokine-mediated mechanisms (68). NK cell-derived IL-12 and membrane-bound CD40L directly promote B cell proliferation and immunoglobulin class switching, while NKp46 engagement with B cell surface ligands induces apoptosis of malignant or dysfunctional B cell clones—a quality control mechanism maintaining immune homeostasis (69) (**Figure 3B**).

### 5.3 Bidirectional crosstalk between B cells and tumor-associated macrophages

The functional interplay between B cells and TAMs critically shapes the immunomodulatory landscape of the TME (70). For instance, in melanoma (71)and breast cancer (72), subcapsular sinus macrophages, particularly CD169^+^ subsets, act as tumor suppressors by inhibiting B cell activation, possibly through forming a physical barrier that restricts B cell activity (71, 73, 74). However, in breast cancer, this regulation is subtype-specific: while Subcapsular sinus macrophages (72)and CD169^+^ TAMs (75) suppress B cell activation, CD169^+^ monocyte-derived macrophages (75) promote B cell activation, highlighting the functional diversity of macrophages in TME. Additionally, macrophage-derived factors like BAFF and APRIL (76–80), which drive B cell proliferation via NF-κB signaling, may contribute to the expansion of malignant B cells in lymphomas (81).

Conversely, B cells actively shape macrophage functions to favor tumor progression through multiple mechanisms. In diffuse large B-cell lymphoma, B cells secrete CCL8, which binds to CCR1/2/3/5 on macrophages, inducing M2 polarization and limiting anti-tumor immunity (82). In mantle cell lymphoma, B cell-derived CCL3 forms a positive feedback loop with M2 macrophages: CCL3 promotes M2 polarization, and M2 macrophages secrete IL-10 to further stimulate CCL3 secretion by MCL cells, accelerating tumor growth (83). B cells also evade macrophage-mediated phagocytosis via pathways like CD47/SIRPα (84) and PD-L1/PD-1 (85), where CD47 on malignant B cells binds SIRPα on macrophages, and PD-L1 interacts with PD-1 on macrophages, both inhibiting phagocytic activity. Furthermore, B cell-derived GABA induces monocyte differentiation into M2 macrophages, which secrete IL-10 to support tumor survival (86). Besides, B cell-derived cytokines, including interferon-γ and granulocyte-macrophage colony-stimulating factor, drive macrophage polarization toward an immunostimulatory M1 phenotype characterized by enhanced tumoricidal activity through nitric oxide production and pro-inflammatory cytokine secretion (87) (**Figure 3C**).

### 5.4 Direct and indirect interactions between B cells and cancer cells

B cells engage in both direct and indirect crosstalk with tumor cells, significantly influencing tumor progression and immune surveillance. A key direct mechanism involves BCR-mediated recognition of TAAs, which can initiate specific activation, proliferation, and antibody production. This antigen-specific engagement may also trigger direct cytotoxic effects against tumor cells via Fas-FasL and granzyme-perforin pathways under certain conditions (88–90) (**Figure 3D**).

Indirectly, B cells modulate tumor behavior through cytokine networks. For instance, B cell-derived lymphotoxin and TNF-α can promote cancer cell survival and invasion by activating NF-κB signaling in malignant cells (91) (**Figure 3D**). Conversely, certain B cell subsets produce anti-angiogenic cytokines that inhibit tumor vascularization (88).

## 6 The role of B cells in tumor immunobiology across cancer types

The dual roles of B cells are not uniformly exhibited across all malignancies; rather, they are shaped by tumor-intrinsic factors, microenvironmental cues, and host immune status. The following section examines how these mechanisms manifest in a cancer-type-specific manner, highlighting both common themes and context-dependent variations.

### 6.1 Hematologic malignancies

In B cell-derived lymphomas, malignant transformation arises from dysregulated proliferation, differentiation, and apoptotic pathways intrinsic to B cell development (92). Within the lymphoma microenvironment, bidirectional crosstalk occurs between neoplastic B cells and residual normal B lymphocytes (93). Tumor cells subvert neighboring B cells through IL-6/STAT3 signaling and CD40L-mediated activation, reprogramming them into pro-tumor effectors that secrete survival factors (94).

Clinically, B cell-targeted therapies demonstrate remarkable efficacy: anti-CD20 monoclonal antibodies (e.g., rituximab) induce complement-dependent lysis in CD20+ lymphomas, while B-cell maturation antigen -directed CAR-T cells and bispecific antibodies achieve deep responses in refractory myeloma by eradicating malignant plasma cell clones (95, 96).

### 6.2 Solid tumors

#### 6.2.1 Melanoma

B cells exhibit dual roles in the immune regulation of melanoma, with their functions critically dependent on subset heterogeneity and microenvironmental features (67, 97–100). On one hand, B cells can enhance antitumor immunity by forming TLSs in collaboration with T cells (97). These structures are enriched with MBCs and activated T cells, significantly improving the efficacy of immune checkpoint blockade (ICB) and correlating with better patient survival (97, 98). Concurrently, clonal expansion, antibody diversity, and affinity maturation of TIL-Bs suggest their involvement in localized antitumor responses, particularly through IgG subclass antibodies that may suppress tumor progression (97). On the other hand, specific B cell subsets exert immunosuppressive effects by secreting IL-10 or upregulating PD-L1, thereby promoting tumor growth (67, 101). Notably, PD-L1^+^ naïve-like B cells directly inhibit T cell function, and their abundance is positively associated with advanced melanoma bone metastasis and poor prognosis (102). Additionally, peripheral blood B-cell levels serve as a predictive biomarker for anti-PD-1 therapy response, with lower B-cell levels correlating with longer survival (103). This functional heterogeneity is closely linked to the balance of B-cell subsets, spatial organization (e.g., infiltrative patterns of activated B-cell follicles in metastatic lymph nodes), and autoimmune-like features, highlighting that targeting specific B-cell subsets or disrupting their immunosuppressive signaling may represent novel therapeutic strategies for melanoma (100).

#### 6.2.2 NSCLC

B cells play a critical immunomodulatory role in NSCLC through the formation of TLSs and functional heterogeneity (104). TLS, serving as hubs for adaptive immune responses, coordinate B and T cell interactions, and their presence significantly enhances the efficacy of PD-L1 inhibitors (e.g., atezolizumab), correlating with prolonged patient survival independently of CD8^+^ T cell signals (104). B cell subsets exhibit dynamic functional divergence: naïve-like B cells suppress tumor growth in early stages by secreting inhibitory factors but diminish in advanced NSCLC, correlating with poor prognosis, while plasma-like B cells exert antitumor activity in early stages but may promote tumor progression in advanced disease via secretion of specific immunoglobulins (e.g., IgG subclasses) or microenvironmental interactions (104, 105). Additionally, B cells generate antitumor effects through antibodies targeting endogenous retroviruses, which are amplified during ICB therapy and enhance therapeutic responses via CXCL13-dependent TLS formation (106). The heterogeneity of B and PCs is influenced by smoking status and TME, with their functional phenotypes (e.g., immunosuppressive plasma cell profiles) predicting postoperative outcomes and ICB efficacy (105). Collectively, strategies targeting TLS formation (e.g., CXCL13 therapy), balancing B cell subsets, or modulating antibody responses (e.g., enhancing anti-Endogenous retrovirus activity) may represent novel therapeutic avenues for NSCLC immunotherapy (106, 107).

#### 6.2.3 Breast cancer

B cells play multiple critical roles in the immune regulation of breast cancer, with their diverse functional subsets and dynamic changes significantly influencing antitumor immune responses and therapeutic outcomes (108). Studies demonstrate that B cells aggregate via TLSs and interact with T follicular helper cells to promote antibody production and T cell activation, constituting a core mechanism underlying responses to ICBs (6). Single-cell sequencing reveals substantial heterogeneity in TIL-Bs, where follicular B cell subsets and chemotherapy-induced inducible T-cell costimulator ligand (ICOSL)^+^ B cell subsets are closely associated with immunotherapy and chemotherapy efficacy, respectively (6). Additionally, B cell infiltration exhibits a synergistic relationship with tumor-associated neutrophils, particularly in TLS-low groups, highlighting the complexity of microenvironmental interactions. Mechanistically, B cell function is regulated by complement signaling (e.g., complement receptor 2/CD55 pathways), as chemotherapy-induced immunogenic cell death drives ICOSL^+^ B cell differentiation via complement-complement receptor 2 signaling, thereby enhancing the effector-to-regulatory T cell ratio to potentiate antitumor immunity (109). These findings not only underscore the therapeutic potential of targeting B cells (e.g., CD23 as a TLS biomarker) but also provide a theoretical foundation for combinatorial immunotherapeutic strategies (e.g., targeting ICOSL or complement pathways).

#### 6.2.4 Renal cell carcinoma

B cells exhibit complex and dynamic dual roles in the immune regulation of renal cell carcinoma (110). On one hand, intratumoral TLSs host B cells that differentiate into antibody-secreting PCs (e.g., IgG/IgA-producing subtypes), mediating antibody-dependent antitumor effects and correlating with improved clinical responses and survival in patients treated with ICBs (111). Spatial transcriptomics reveals that B cells within TLS undergo clonal diversification, expansion, and migration, with their maturation status and localization (e.g., tumor-proximal mature TLS containing CD23^+^ GCs) significantly influencing prognosis. Mature TLS are associated with favorable survival outcomes, while immature tumor-distal TLS are enriched with immunosuppressive cells (e.g., PD-L1^+^ macrophages and regulatory T cells), reflecting microenvironmental heterogeneity (111). On the other hand, dynamic shifts in B cell subsets are linked to treatment efficacy and toxicity: combined ICB therapy promotes differentiation of circulating B cells into memory phenotypes (e.g., increased switched MBCs correlate with therapeutic efficacy), whereas plasmablast expansion is associated with severe immune-related adverse events (e.g., hypophysitis) (112). Notably, high TIL-Bs may recruit M2 macrophages and Tregs, exacerbate T-cell exhaustion (marked by upregulation of PD-1/CTLA-4/TIM-3), and diminish the efficacy of combination therapies (e.g., anti-PD-1 inhibitors combined with axitinib) (113). These findings underscore the dual nature of B cells in RCC—balancing antitumor potential and immunosuppressive risks—with their functional polarization shaped by spatial distribution, maturation status, and therapeutic interventions, offering critical insights for precision immunotherapy strategies.

#### 6.2.5 Hepatocellular carcinoma

The role of B cells in HCC is dualistic and complex, encompassing both antitumor immune regulation and tumor-promoting mechanisms through specific subsets or signaling pathways (114–116). Studies indicate that B cells collaborate with T cells to suppress HCC progression, such as by forming TLSs to enhance T cell memory function or via B-cell-related gene models that predict immunophenotypes and prognosis, highlighting their antitumor potential (117). However, B cells can also drive immunosuppression through distinct mechanisms, such as IL-21 receptor signaling inducing immunosuppressive IgA^+^ B cells to inhibit CD8^+^ T cell activity, or Ten-eleven translocation methylcytosine dioxygenase 2-mediated IL-10^+^ regulatory B cells (Breg) promoting tumor immune evasion (118). Additionally, interactions between B cells and innate lymphoid cells, exemplified by ICOSL signaling, exacerbate inflammatory microenvironments and accelerate HCC progression (115). Spatial dynamics analysis further reveals that colocalization patterns of B and T cells (e.g., TLS or lymphoplasmacytic microenvironments) significantly influence clinical outcomes and immunotherapy responses (119). Molecular regulatory mechanisms within the TME, such as Pre-mRNA processing factor 19-mediated degradation of DEAD-box helicase 5 suppressing B cell recruitment or dysregulation of the CXCL12/CXCR4 axis, alongside epigenetic modifications (e.g., Ten-eleven translocation methylcytosine dioxygenase 2-dependent IL-10 expression), reshape B cell functionality and emerge as potential therapeutic targets (120, 121). In summary, B cells exhibit functional heterogeneity in HCC, with their pro- or antitumor effects determined by subset characteristics, spatial distribution, and microenvironmental signaling networks. Targeting B cell-related pathways (e.g., ICOS, IL-21R, Ten-eleven translocation methylcytosine dioxygenase 2) or combining therapies with ICBs may offer novel strategies for personalized immunotherapy in HCC.

#### 6.2.6 Colorectal cancer

B cells in CRC exhibit functional diversity and microenvironment dependency, contributing to both antitumor immune regulation and pro-tumor mechanisms (122–124). Studies show that B cell subsets such as TLS-associated CD20+ B cells and IgG PCs collaborate with CXCL13^+^ CD8^+^ T cells to promote TLS formation, enhance antigen presentation, and correlate positively with high microsatellite instability, high tumor mutation burden, and immunotherapy response, indicating their antitumor potential (122). However, specific B cell subsets, such as leucine-tRNA synthase 2-expressing B cells, suppress antitumor immunity through TGF-β1-dominant regulatory features (123). These cells are driven by leucine metabolism and rely on mitochondrial nicotinamide adenine dinucleotide^+^ regeneration and sirtuin 1 signaling, promoting CRC immune evasion (123). Spatial heterogeneity analysis reveals distinct B cell developmental trajectories and CD20^+^ B cell abundance between right- and left-sided CRC, with CD20^+^ B cell enrichment in right-sided CRC predicting favorable prognosis, while their depletion impairs anti-PD-1 therapy efficacy (124). Additionally, microbiota-immune interactions regulate B cell function: specific bacteria like *Helicobacter hepaticus* induce follicular helper T cells to promote TLS maturation and antitumor immunity, whereas *Alcaligenes faecalis* suppresses IgA^+^ B cell homing and disrupts the intestinal barrier via acetate-mediated vinculin acetylation, driving the inflammation-to-cancer transition (11, 125). Notably, activated B cells inhibit CRC liver metastasis via the SDF-1-CXCR4 axis, yet their depletion in metastatic sites correlates with increased metastasis, while immature plasma cell subsets are linked to metastasis progression (126). In summary, B cells in CRC exhibit a dual role, with their function determined by subset characteristics, metabolic states, spatial localization, and dynamic interactions with microbiota and T cells. Targeted modulation of B cell subsets—such as inhibiting leucine-tRNA synthase 2-expressing B cells, enhancing CD20^+^ B cells, or promoting follicular helper T-B cell collaboration—may provide novel strategies for CRC immunotherapy.

### 6.3 Analysis of similarities and differences in B cell function across tumor types

In the same tumor, the dual antitumor and pro-tumor roles of B cells arise from the integration of multiple interconnected mechanisms. Firstly, B cell subset heterogeneity is foundational: antitumor subsets, such as GC B cells and PCs, exert protective effects by producing high-affinity antibodies and forming TLSs to recruit and activate T cells (127). In contrast, pro-tumor subsets like Bregs secrete immunosuppressive cytokines (e.g., IL-10, TGF-β) to inhibit effector T/NK cells and promote Treg differentiation, while exhausted or aged B cells impair antigen presentation and express high levels of immune checkpoints (e.g., PD-1, TIM-1) to exhaust T cells (128).

Secondly, the TME actively shapes this duality through metabolic and signaling regulation (129). Metabolic stress, including nutrient competition with tumor cells and hypoxia, induces hypoxia-inducible factor-1α and impairs B cell effector functions (e.g., reduced antibody production) (129). Meanwhile, TME-derived signals selectively suppress antitumor B cells while recruiting or activating Bregs (130).

Thirdly, heterogeneous expression of immune checkpoint molecules contributes: pro-tumor subsets (Bregs, exhausted B cells) upregulate PD-L1, CTLA-4, etc., to transmit inhibitory signals, whereas antitumor subsets retain low checkpoint expression to maintain functionality (131).

Lastly, clonal origin and antigen specificity differences underpin functional divergence. B cell clones recognizing TAAs or neoantigens differentiate into antitumor PCs, while those targeting self-antigens or non-specific inflammatory signals are prone to becoming Bregs (132). These clones compete for resources and signals in the TME, further amplifying functional heterogeneity.

Collectively, these mechanisms—subset diversity, TME regulation, checkpoint heterogeneity, and clonal specificity—synergistically drive the coexistence of B cells’ contradictory roles within the same tumor.

The functional heterogeneity of B cells across different cancer types underscores the necessity of understanding their roles in a context-specific manner. This knowledge not only enhances our comprehension of tumor immunobiology but also informs the development of tailored therapeutic strategies. In the following section, we explore how B cells influence—and can be harnessed to improve—current cancer treatments, including ICBs, cancer vaccines, and chemotherapy.

## 7 Comparative analysis of B cell functions in human and murine systems

B cells in both humans and mice play dual and multifaceted roles in tumor immunity, contributing to both anti-tumor and pro-tumor responses through conserved mechanisms. In both species, B cells are involved in antigen presentation, antibody production, and the formation of TLSs, which support T-cell activation and adaptive immunity. For example, in human cancers such as breast and lung adenocarcinoma, as well as in mouse models like 4T1 and CT26, B cells enhance antitumor immunity by producing antibodies, facilitating antigen cross-presentation, and promoting cytotoxic T-cell responses (2). Bregs also exist in both systems and suppress immune activity through cytokines such as IL-10 and TGF-β, highlighting a shared functional dichotomy in B cell responses (133, 134).

However, important species-specific differences are evident. In humans, Bregs are commonly identified by the phenotype CD19^+^CD24^hi^CD38^hi^ (135), particularly in gastric and pancreatic cancers, whereas murine Bregs are often characterized as CD19^+^CD5^+^CD1D^hi^ (136). Additionally, therapeutic outcomes can diverge; anti-CD20 therapy effectively depletes B cells in some human cancers but may exacerbate tumor growth in mice due to the expansion of CD20^low^ Breg subsets (137, 138). Human B cells also display greater heterogeneity and spatial complexity within tumors, frequently including distinctive subsets such as double-negative (CD27^−^IgD^−^) MBCs and dense plasma cell infiltrates, whose roles are not fully paralleled in mouse models (139). These differences underscore the necessity of cautiously translating murine findings to human applications and combining insights from both systems to advance cancer immunotherapy.

## 8 The impact of B cells on tumor immunotherapy and chemotherapy

Building upon the mechanistic insights into B cell functions in tumor immunity, we now turn to their clinical implications, particularly in the context of immunotherapy and chemotherapy, where B cells serve as both mediators of response and potential therapeutic targets.

### 8.1 Immune checkpoint inhibitor therapy

**Figure 4:**
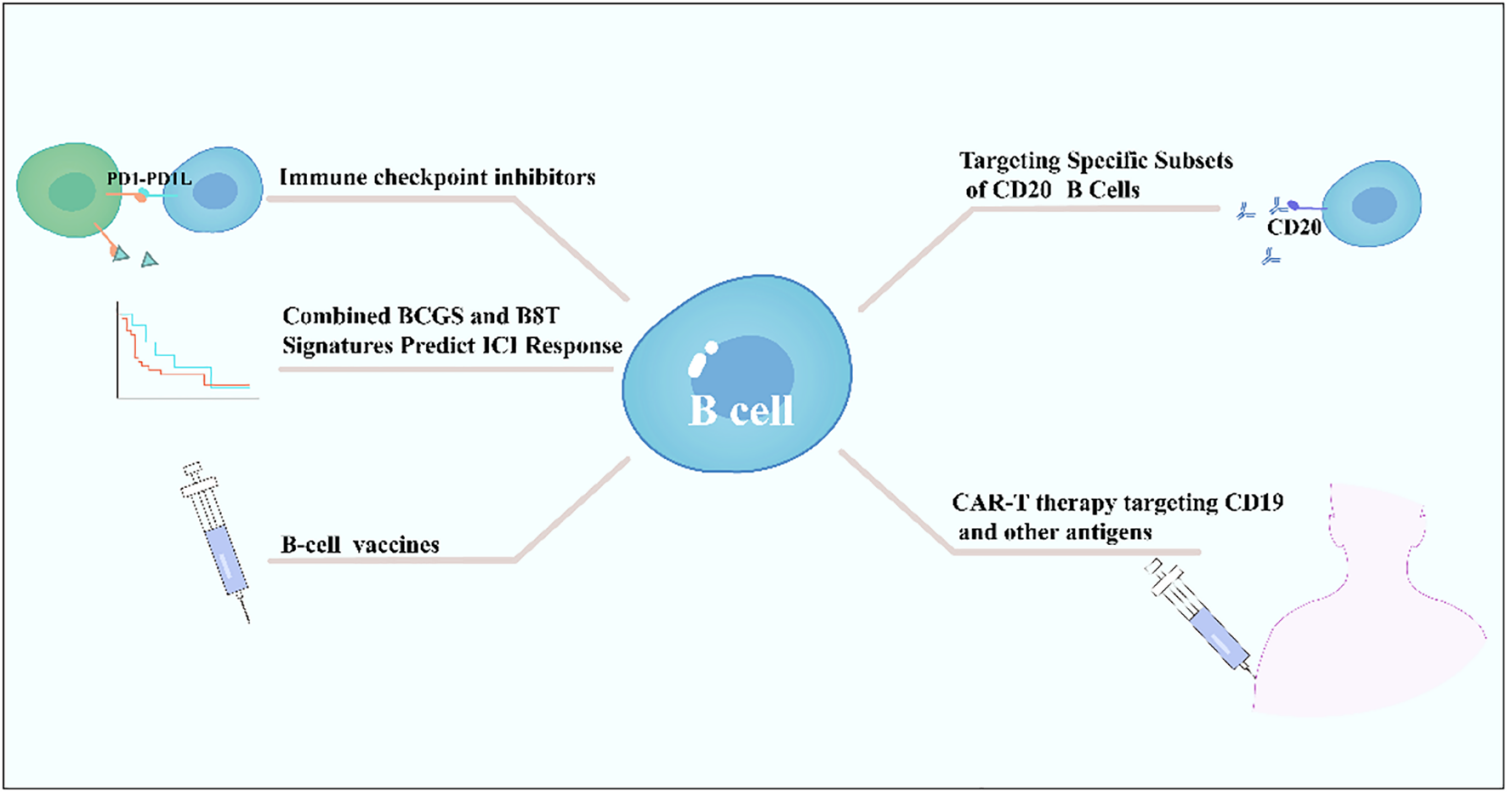
B cells on tumor immunotherapy.

B cells exhibit dual roles in immune checkpoint inhibitor therapy, with their functions and predictive value varying by tumor type and immune microenvironment. In solid tumors such as bladder cancer, a high intratumoral B-cell gene signature combined with CD8^+^ T-cell signals significantly predicts ICB efficacy, correlating with prolonged overall survival particularly in male patients, while no such association is observed in females, suggesting sex-specific immune regulatory mechanisms (140). In B-cell lymphomas, HLA-dependent antigen presentation defects—such as class II-associated invariant chain peptide retention caused by HLA-DM deficiency—constitute a key immune escape mechanism, impairing tumor antigen presentation and potentially diminishing ICB efficacy. Notably, abnormal HLA expression patterns occur in 62-88% of cases in classic Hodgkin lymphoma and diffuse large B-cell lymphoma (141). Additionally, combination strategies targeting B-cell surface molecules (e.g., CD20-targeting antibodies) with ICBs have emerged as a research focus. Bibliometric analyses highlight CAR-T therapy, next-generation CD20 antibodies, and PD-1/PD-L1 inhibitors as emerging directions, potentially overcoming treatment barriers in B-cell lymphomas by modulating B-cell functions or enhancing T-cell activity (142). In summary, B cells serve not only as potential predictive biomarkers for ICB efficacy (e.g., B8T signature) but also as critical targets for therapeutic optimization (e.g., CD20-targeted combinations) and immune escape mechanism analysis (e.g., HLA abnormalities). Their multifaceted roles necessitate comprehensive evaluation based on tumor type, sex, and molecular characteristics (140–142) (**Figure 4**).

### 8.2 Cancer vaccines

B cells play a critical role in cancer vaccine development as key effector cells, enhancing antitumor responses through the activation of humoral immunity and synergistic action with adaptive immunity (143). B-cell epitope vaccines, such as HER-vaxx, target linear epitopes of TAAs (e.g., Her-2/neu) to induce polyclonal, high-affinity antibodies (144). Their efficacy rivals that of monoclonal antibodies (e.g., trastuzumab) while offering advantages such as lower cost, reduced toxicity, and controllable half-lives (145). For instance, a CD19-targeted fusion protein (scFv-Her2D4) combined with PD-1 antibodies reverses T-cell exhaustion, reduces MDSC/Treg infiltration, and promotes complete tumor regression (146) (**Figure 4**).

To enhance immunogenicity, vaccine design employs multivalent antigen strategies (e.g., bivalent antigens), which promote GC B cell differentiation and long-lived plasma cell generation through B-cell receptor (BCR) crosslinking, significantly boosting antibody levels (147). Antigen delivery systems targeting DC surface markers (e.g., CD11c, Xcr1) or MHC II molecules further optimize antigen-presenting cell (APC) presentation efficiency (147).

Synergy between B-cell and T-cell vaccines is achieved through coordinated mechanisms: CD8^+^ T cells directly lyse tumors via pMHC recognition, while B cells, with CD4^+^ T-cell assistance, secrete antibodies mediating antibody-dependent cellular cytotoxicity or blocking immunosuppressive signals (e.g., PD-1/PD-L1) (148). Additionally, active immunization strategies using immune checkpoint mimotopes (e.g., PD-1 mimotopes) induce endogenous antibodies to block inhibitory pathways (143). When combined with tumor-specific vaccines (e.g., Her-2), this approach significantly enhances efficacy, as demonstrated in preclinical models by reduced tumor proliferation and induced apoptosis (143) (**Figure 4**).

Novel vaccine platforms integrate chemical modifications (e.g., fluorinated cyclic dinucleotides to enhance STING activation), nanocarriers (e.g., black phosphorus nanosheets), and dynamic APC behavior regulation (e.g., DNA hydrogels) to achieve multimodal immune activation (148). Future directions focus on optimizing multivalent antigen design, APC-targeted delivery systems, and B/T cell dual pathway synergy to overcome clinical translation barriers, advancing cancer immunotherapy toward high-efficiency, precision-driven multidimensional strategies (147).

### 8.3 B cell dynamics during chemotherapy

Chemotherapy exerts profound and selective effects on B cell subsets, altering their proportions, differentiation trajectories, and functional phenotypes in ways that impact antitumor immunity. Notably, chemotherapy often increases the proportion of naive B cells (e.g., IgD+CD27- subsets) while depleting MBCs, with incomplete recovery of MBCs even years post-treatment (149). Transitional B cells rebound rapidly after chemotherapy, exceeding baseline levels temporarily, whereas follicular and marginal zone B cells show prolonged depletion, particularly marginal zone B cells, which are critical for rapid antibody responses to T-independent antigens (149–152). PCs exhibit relative resistance to chemotherapy-induced depletion compared to other B cell subsets, with preserved or enhanced antibody secretion—including IgG targeting TAAs—facilitated by chemotherapy-induced antigen release and immune complex formation (153). Bregs are selectively reduced by certain chemotherapeutics, with decreased IL-10 secretion and increased apoptosis, potentially alleviating immunosuppression and enhancing treatment efficacy (154).

These dynamic changes in B cell subsets are tightly linked to treatment responses. For instance, post-chemotherapy increases in ICOSL+ B cells correlate with elevated effector T cell/Treg ratios and improved survival in breast cancer (109), while class-switched MBCs with upregulated CD86 predict better responses to platinum-based therapy in ovarian cancer (155). Clonal expansion patterns also shift: chemotherapy drives naive B cell differentiation into antigen-specific MBCs and PCs, with clonal diversity linked to robust antitumor antibody responses (149). Additionally, chemotherapy modulates B cell-T cell crosstalk—via CD86 upregulation on B cells or ICOSL-ICOS interactions—to enhance T cell activation, further shaping treatment outcomes (156). Together, these dynamics highlight B cells as key regulators of chemotherapy efficacy, with subset-specific changes offering potential biomarkers and therapeutic targets.

### 8.4 Clinically approved B cell-targeted therapies in solid tumor

In solid tumor therapy, several B cell-targeted strategies have been applied in clinical practice or clinical trials. Monoclonal antibodies (mAbs) represent a key approach. Rituximab, an anti-CD20 mAb, has been used to deplete B cells in certain solid tumors; for example, it inhibits cross-talk between B cells and TAMs in pancreatic ductal adenocarcinoma, showing potential in preclinical and early clinical settings (157). Ibrutinib, a Bruton’s tyrosine kinase inhibitor, has also been explored for B cell depletion in pancreatic ductal adenocarcinoma, aiming to reduce the pro-tumorigenic effects of Bregs (157). Additionally, anti-CD20 mAbs have been tested in melanoma, with studies indicating that depleting tumor-associated B cells may improve outcomes in patients at high risk of recurrence (158, 159).

Vaccines and adjuvants targeting B cells to enhance anti-tumor immunity are another active area. HER-2/neu-based vaccines, such as those using GM-CSF as an adjuvant, have been evaluated in breast cancer to boost anti-HER2/neu antibody responses (160). A Phase Ib trial of the B cell epitope vaccine IMU-131/HER-Vaxx in HER-2+ gastroesophageal adenocarcinoma demonstrated dose-dependent increases in HER2/neu-specific IgG levels (161). Adjuvants like CpG oligodeoxynucleotides and monophosphoryl lipid A have been combined with tumor antigens in vaccines for melanoma, non-small cell lung cancer, and prostate cancer, enhancing cytotoxic antibody functions and inducing IgG production (162, 163).

## 9 Future perspectives

While significant progress has been made in unraveling the roles of B cells in tumor biology, critical challenges and knowledge gaps remain. Future studies should prioritize elucidating the functional heterogeneity of B-cell subsets within the TME, delineating their precise mechanisms in tumorigenesis, therapeutic response, and immune regulation. This includes deciphering complex crosstalk between B cells and other immune components (e.g., T cells, DCs) and identifying microenvironmental factors (e.g., cytokines, metabolic cues) that modulate these interactions. Concurrently, advancing targeted B-cell therapies requires optimizing specificity to suppress pro-tumor subsets while preserving antitumor functions, alongside developing combination strategies with checkpoint inhibitors or chemotherapy to minimize toxicity and enhance therapeutic efficacy. Translational efforts must focus on identifying robust biomarkers—such as B-cell receptor diversity or TLS signatures—to predict treatment outcomes and guide personalized immunotherapy. Additionally, emerging mechanisms involving non-coding RNAs, metabolic reprogramming, and exosome-mediated communication between B cells and tumors warrant deeper exploration for their diagnostic and therapeutic potential. By addressing these priorities, a deeper understanding of B-cell biology in oncology will pave the way for innovative immunotherapies, ultimately improving survival and quality of life for cancer patients.
